# Improving *Saccharomyces cerevisiae* ethanol production and tolerance via RNA polymerase II subunit Rpb7

**DOI:** 10.1186/s13068-017-0806-0

**Published:** 2017-05-15

**Authors:** Zilong Qiu, Rongrong Jiang

**Affiliations:** School of Chemical and Biomedical Engineering, Nanyang Technological University, 62 Nanyang Drive, Singapore, 637459 Singapore

**Keywords:** Global transcription machinery engineering (gTME), Transcriptional engineering, RNA polymerase II, Subunit Rpb7, Ethanol tolerance, Oxidative tolerance, VHG fermentation, Ethanol titers, Ethanol productivity

## Abstract

**Background:**

Classical strain engineering methods often have limitations in altering multigenetic cellular phenotypes. Here we try to improve *Saccharomyces cerevisiae* ethanol tolerance and productivity by reprogramming its transcription profile through rewiring its key transcription component RNA polymerase II (RNAP II), which plays a central role in synthesizing mRNAs. This is the first report on using directed evolution method to engineer RNAP II to alter *S. cerevisiae* strain phenotypes.

**Results:**

Error-prone PCR was employed to engineer the subunit Rpb7 of RNAP II to improve yeast ethanol tolerance and production. Based on previous studies and the presumption that improved ethanol resistance would lead to enhanced ethanol production, we first isolated variant M1 with much improved resistance towards 8 and 10% ethanol. The ethanol titers of M1 was ~122 g/L (96.58% of the theoretical yield) under laboratory very high gravity (VHG) fermentation, 40% increase as compared to the control. DNA microarray assay showed that 369 genes had differential expression in M1 after 12 h VHG fermentation, which are involved in glycolysis, alcoholic fermentation, oxidative stress response, etc.

**Conclusions:**

This is the first study to demonstrate the possibility of engineering eukaryotic RNAP to alter global transcription profile and improve strain phenotypes. Targeting subunit Rpb7 of RNAP II was able to bring differential expression in hundreds of genes in *S. cerevisiae*, which finally led to improvement in yeast ethanol tolerance and production.

**Electronic supplementary material:**

The online version of this article (doi:10.1186/s13068-017-0806-0) contains supplementary material, which is available to authorized users.

## Background

In breeding of strains with robustness under industrial conditions and high production capacities of desired-compounds, one major challenge is that cellular phenotypes are often regulated by hundreds of genes, which makes it difficult for conventional engineering methods to achieve desirable expression profile simultaneously. To address the complexity of eliciting optimal expression profile for desired phenotype, engineering strategies call for spontaneous modulation of global gene expression and metabolism shifts [30]. In recent years, engineering components of global transcription machinery has been explored to fulfill the requirement of fine-tuning or reprogramming microbial cellular transcription profile. In prokaryotic microbes, a few key regulators have been successfully engineered to alter *Escherichia coli* (*E. coli*) and *Zymomonas mobilis* phenotypes, including sigma factor σ^70^ [2, 45], alpha subunit of RpoA [27], exogenous regulator IrrE [9], global regulator Hha & H-NS [21, 22], cAMP receptor protein (CRP) [11, 53]. In eukaryotic microbes, the transcriptional machinery is more complex, with a large set of general and specific transcription factors involved [15]. Only TATA-binding protein (Spt15) [1, 31], TATA-binding protein-associated factor Taf25 [54], and zinc finger-containing artificial transcription factors [38] have been successfully engineered to alter *Saccharomyces cerevisiae* (*S. cerevisiae*) phenotypes so far. All these approaches focus on engineering a specific transcription factor (TF) to alter DNA-binding specificity and thus change global gene expression.

**Fig. 1:**
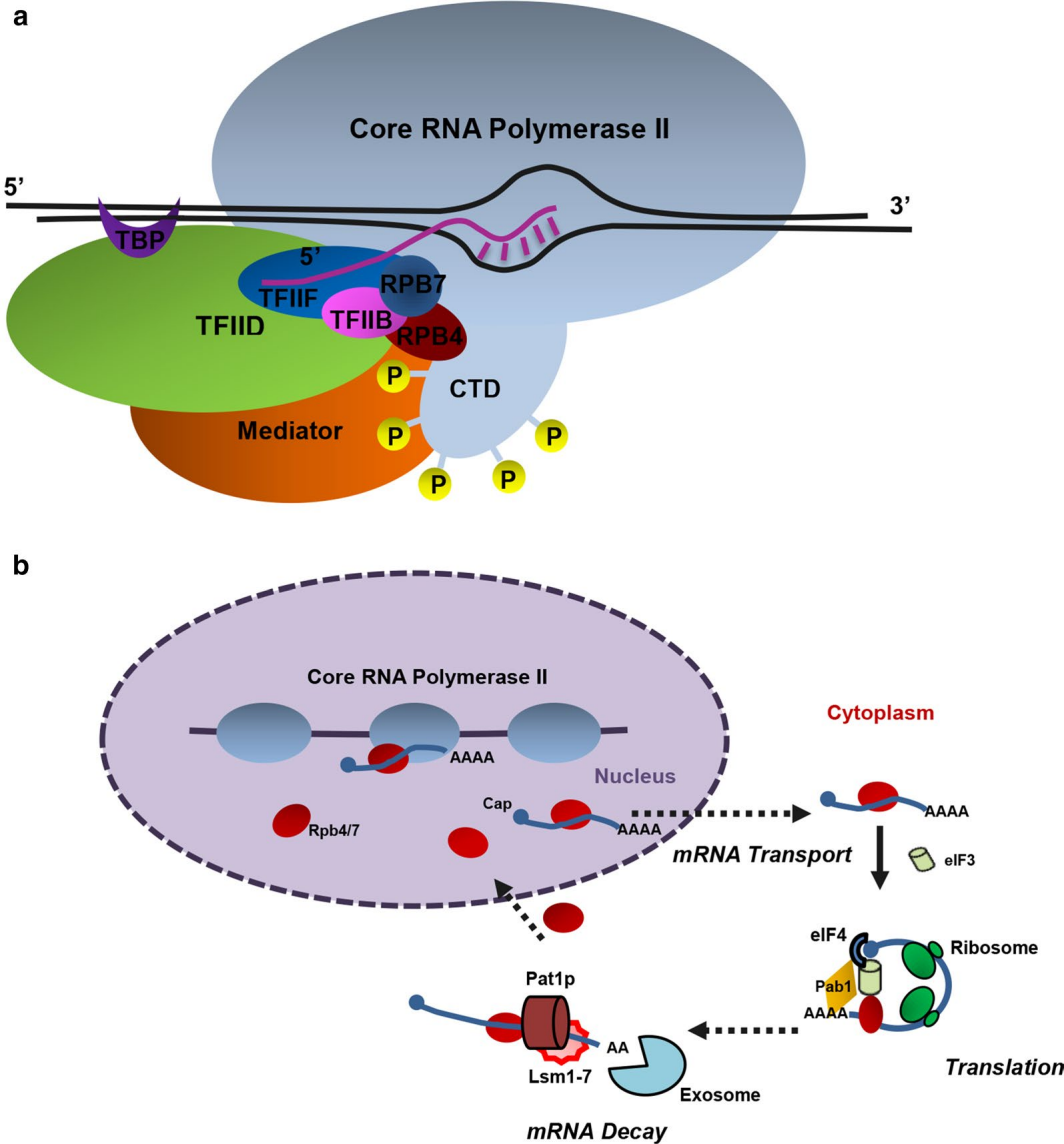
Multifunction of Rpb7 in gene expression. Rpb7 usually fulfills its function by forming sub-complex with Rpb4, but the major role of Rpb4 is to augment the interaction of Rpb7 with Pol II [42]. **a** In the transcription initiation complex, the Rpb4/7 sub-complex is situated closed to the nascent-transcript-exit groove and adjacent to Rpb1 C-terminal domain (CTD) linker region [10], and it is also located near general transcription factor TFIIB and can physically interact with TFIIF [43]. **b** The role of Rpb4/7 in post-transcription regulation, including mRNA export, translation, and mRNA decay [19]

Apart from TFs, RNA polymerase II (RNAP II) plays a central role in synthesizing all mRNAs. It is the core enzyme of yeast global transcription machinery, which not only interacts with DNA, transcript RNA, and regulatory proteins during mRNA synthesis, but also is involved in mRNA post-initiation regulation [4, 28, 40]. The fine-tuning of the subunits of RNAP II may also have the potential to induce perturbations on global transcription. In this work, instead of engineering a specific TF from *S. cerevisiae*, we tried to target RNA polymerase II subunit Rpb7 to improve yeast ethanol tolerance and production. Among the twelve subunits of RNAP II, Rpb7 serves as an ‘mRNA coordinator’ [19] at different stages of genes expression, including (i) interacting with processing factors of RNAP II transcription apparatus and nascent RNA transcripts [24, 52]; and (ii) participating in mRNA export and decay [32] (Fig. 1). The multifunction of Rpb7 suggests the possibility of mutating Rpb7 to elicit cellular transcription profile change and achieve desired phenotypes in yeast.

As for bioethanol industry, ethanol inhibition during the production stage is one of the major causes that lead to decreased ethanol production and stuck fermentation [18, 47, 55]. The presence of high concentration ethanol may denature cellular protein, alter plasma membrane permeability, and inhibit mitochondrial function, which could slow down glucose transport and eventually inhibit yeast growth and ethanol fermentation [3, 34]. Previous works have successfully demonstrated the possibility of enhancing yeast ethanol production by improving its ethanol tolerance primarily. For example, the introduction of *TPS1* (6-phosphate-trehalose synthase) from *Saccharomycopsis fibuliger* in *S. cerevisiae* resulted in better survival in 18% (v/v) ethanol and ~15% increase of final ethanol concentration [8]. Inhibiting *ATH1* (acid trehalase) expression promoted yeast growth in 8% (v/v) ethanol and ~100% increase in ethanol productivity [25]. An ethanol-tolerant mutant generated from UV-C mutagenesis displayed ~18% more ethanol accumulation than the wild-type [46]. Based on the presumption that improved ethanol resistance would lead to enhanced ethanol production in yeast, in this work, the ethanol tolerance trait was chosen as the primary engineering target to isolate efficient ethanol producing strain.

Our group has successfully improved *E. coli* ethanol tolerance by engineering its global regulator cAMP receptor protein (CRP) before [12]. Here, random mutagenesis library of Rpb7 was constructed and subjected to screening under ethanol stress. The isolated variant with elevated ethanol tolerance had also shown much enhanced ethanol titers during very high gravity (VHG) laboratory fermentation as compared to the control. Fermentation was further investigated, and genome-wide DNA microarray analysis was performed to reveal cellular transcription profile change. To the best of our knowledge, this study is the first to demonstrate the possibility of engineering eukaryotic RNAP to alter global transcription profile and hence improve strain phenotypes.

## Methods

### Strains and media

*Escherichia coli* DH5α (Invitrogen, San Diego, USA) was used for cloning and cultured at 37 °C in Luria–Bertani (LB) medium (bacto tryptone 10 g/L, yeast extract 5 g/L, sodium chloride 10 g/L). *S. cerevisiae* BY4742 and CEN.PK2-1C were purchased from EUROSCARF (Frankfurt, Germany) and cultured in YPAD media (20 g/L peptone and 10 g/L yeast extract supplemented with 20 g/L glucose) at 30 °C. Recombinant *E. coli* and yeast strains were screened in LB containing 100 μg/mL ampicillin and YPAD containing 200 μg/mL G418, respectively.

### Plasmid and mutant library construction

Gene *RPB7* was amplified from BY4742 genome using primer 1 and 2 (see Additional file 1: Table S1), and inserted between *Bam*HI and *Xho*I of plasmid pRS41K (Euroscarf, Frankfurt, Germany). Native promoter *RPB7p* was amplified using primer 3 and 4 containing *Sac*I and *Bam*HI site, respectively, and inserted into plasmid pRS41K. *CYCT1* was cut from plasmid p416 MET25 (ATCC^®^87324™) using *Xho*I and *Kpn*I and ligated with plasmid pRS41K. The resulting plasmid harboring *RPB7p*-*RPB7*-*CYCT1* expression cassette was denoted as p41K-*RPB7*

Random mutagenesis library of *RPB7* was generated by error-prone PCR according to GeneMorph^®^ II Random Mutagenesis Kit (Agilent Technologies, CA, USA). Specifically, 30–40 ng DNA template was applied for the amplification of *RPB7* with primer 5 and 6. PCR program was set as 5 min at 95 °C, 30 cycles of 95 °C for 40 s, 55 °C for 45 s, and 72 °C for 1.5 min, followed by 10 min at 72 °C. The PCR products (4 μg) and *Bam*HI & *Xho*I double digested p41K-*RPB7* plasmid (1 μg) were electroporated into BY4742 strain using an Eppendorf^®^ multiporator (Hamburg, Germany) following *Benatuil*’s protocol [7].

### Mutant identification

The mutant library was cultured in 200 mL YPAD medium supplemented with elevated ethanol concentration [from 8 to 12% (v/v)]. After three to five successive subcultures, the enriched cell culture diluted by 10^6^–10^7^ was spread onto YPAD plates. Individual colonies were randomly picked for miniprep [Zymoprep II kit (Zymo Research, Orange, CA, USA)] and sent for DNA sequencing. To eliminate the possibilities of introducing mutations from plasmid backbone and host strain during enrichment selection, mutant *RPB7* gene fragment was digested and re-inserted into a fresh plasmid p41K-*RPB7* backbone and retransformed into fresh BY4742 background, generating mutant M1 used in this study.

### Mutant growth under stress

Overnight cell culture was inoculated into 5 mL fresh YPAD medium containing 0, 8, 10% ethanol (v/v) with an initial OD600 of 0.1. Both the mutant and the control growth were recorded by monitoring their absorbance at 600 nm, respectively. 5 mL YPAD medium supplemented with 3.5 mM H_2_O_2_, 80 mM acetic acid, or 1.5 M NaCl w as used to test mutant oxidative, acetic, and osmotic tolerance, respectively. Inhibitors in lignocellulose hydrolysates, i.e., levulinic acid (196 mM), furfural (1.16 g/L), HMF (17.5 mM), ferulic acid (1 mM), vanillin (13.1 mM), and *p*-coumaric acid (12 mM) were supplemented into 5 mL YPAD medium, respectively, for M1 tolerance test.

### Ethanol fermentation

Both mutant and the control were grown in 50 mL YPAD medium in 200-mL flasks to accumulate large amount of cells for high inoculum fermentation (initial OD 600: 15). During laboratory VHG fermentation, the culture YPAD media was about 2/3 (33/50 mL) of the overall capped test tube volume to achieve micro-aerobic conditions. The initial glucose was 300 g/L. Cell samples were taken every 6 h for OD600 measurement and the supernatant from centrifugation was collected for metabolites analysis described below.

The fermentation process was investigated by altering host strain background, initial glucose concentration (50–300 g/L), and initial pH (5–8).

### Analytical method

The concentration of yeast metabolites was quantified chromatographically by an Agilent 1100 HPLC system equipped with a Refractive Index Detector (RID). Ethanol, glucose, acetic acid, and glycerol were separated using an Aminex HPX-87H Ion Exclusion Column (Bio-Rad, Hercules, USA) at 35 °C, with 5 mM sulfuric acid mobile phase at a flow rate of 0.6 mL/min. All samples with two biological replicates were filtered through a 0.20-μm filter before HPLC analysis.

### DNA microarray and quantitative real-time reverse transcription PCR (qRT-PCR)

Total cellular RNAs were extracted from both the mutant and the control using RNeasy^®^ Mini Kit and RNase-Free DNase Set (Qiagen, Hilden, Germany) under the following two conditions: (i) when cells reached early exponential phase (OD600 ~1.0) in YPAD; (ii) after 12 h VHG fermentation. RNA quality and integrity were verified by gel electrophoresis, as well as by measuring 260/230 ratios with a NanoDrop 1000 spectrophotometer (Thermo Scientific, MA, USA). Two biological replicates of each sample were sent to Genomax Technologies (Singapore) for DNA microarray assay using Yeast (V2) Gene Expression Microarray, 8 × 15 K Microarrays (Agilent technologies, USA). The obtained data were analyzed by Agilent Genespring GX software, and the *p* values were calculated by unpaired Student *t* test.

qRT-PCR was performed using StepOnePlus™ Real-Time PCR System (Applied Biosystems, MA, USA). The isolated RNA described above was reverse transcribed to cDNA by iScript cDNA Synthesis Kit according to manufacturer’s protocols (Bio-Rad, CA, USA) with 500 ng mRNA as template. All primers used for qRT-PCR are listed in Additional file 1: Table S2. qRT-PCR was performed in 20 µL reaction mixture, containing 10 µL 2 × SYBR™ Green master mix (Life Technologies, MA, USA), 2 µL primers (5 µM), 6 µL H_2_O, and 2 µL cDNA. Gene expression level changes were calculated by 2^−ΔΔCt^ method, using 18 s rRNA (*RDN18*) as reference gene.

### Intracellular reactive oxygen species (ROS) level

The intracellular ROS level of mutant and the control was measured using a sensitive probe 2′,7′-dichlorodihydrofluorescein diacetate (H_2_DCFDA). Overnight culture was inoculated into fresh 5 mL YPAD medium until OD600 reached 1.0. Cells were washed twice with 10 mM pH 7.0 potassium phosphate buffer (PPB), re-suspended in 5 mL PPB supplemented with 10 µM H_2_DCFDA, and incubated at 30 °C, 200 rpm for 30 min in darkness. Cells were then lysed by vortex with glass beads (425–600 μm). Cell lysate was applied per well in a 96-well microplate (black background) to measure its fluorescence intensity by a Tecan Infinite 200 microplate reader (Mannedorf, Switzerland) with excitation at 485 nm and emission at 535 nm. The relative fluorescence unit was normalized according to the total protein concentration in cell lysate, measured with Bradford’s reagent in an Eppendorf^®^ Biophomoter (Hamburg, Germany).

## Results

### Mutant isolation

Random mutagenesis library bearing ~10^8^ clones was subjected to enrichment selection, and ~30 individual colonies were randomly picked, sequenced, and their growth performance was tested under ethanol stress. The best mutant M1 was found to have two amino acid mutations, Y25N and A76T. To eliminate the effects of possible mutations from chromosome or plasmid backbone, M1 *RPB7* gene fragment was re-inserted into digested p41K-*RPB7* backbone and retransformed into fresh BY4742 background. The newly transformed strain M1 was used in this study.

**Fig. 2:**
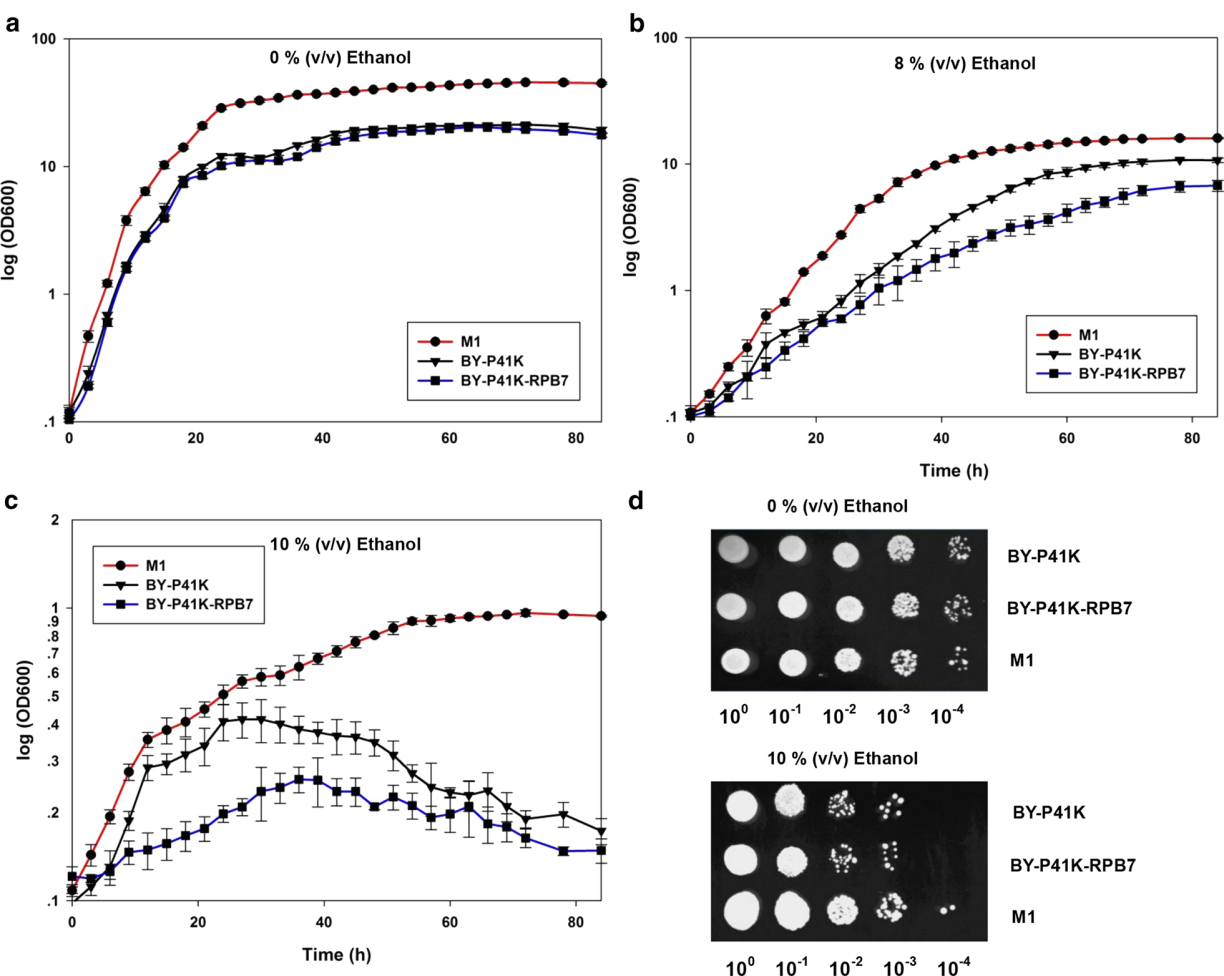
Ethanol tolerance. M1 in **a** 0% ethanol; **b** 8% (v/v) ethanol; **c** 10% (v/v) ethanol. All cells were grown in YPAD medium at 30 °C, 225 rpm. **d** Spot assay. Tenfold serial dilutions of the culture (5 μL) were spotted on YPAD agar with or without 10% (v/v) ethanol. The spotted agar plates were incubated at 30 °C for 2 days

In the absence of ethanol stress, M1 displayed slightly better growth than BY-P41K (BY4742 strain harboring plasmid p41k) and BY-P41K-RPB7 (BY4742 strain harboring plasmid p41K-*RPB7*) (Fig. 2a). With 8% (v/v) ethanol present (Fig. 2b), the growth rate of M1 was 0.427 h^−1^, doubling that of the BY-41K and BY-P41K-RPB7. When ethanol concentration was further increased to 10% (v/v, Fig. 2c), a sub-lethal ethanol environment, M1 displayed a modest growth rate at 0.021 h^−1^, with a prolonged exponential phase, whereas the growth of BY-P41K-RPB7 was completely inhibited. When the native *RPB7* promoter of M1 was replaced by constitutive promoter *TEF1p*, the resulting variant showed no further improvement in 8% (v/v) ethanol as compared with M1, which implied that overexpression of mutant RPB7 might not lead to better ethanol resistance (data not shown). The ethanol resistance of M1 was further exploited through ethanol susceptibility assay on agar plates. As shown in Fig. 2d, M1 exhibited better survival than BY-P41 K and BY-P41K-RPB7 when exposed to 10% (v/v) ethanol. As BY-P41K and BY-P41K-RPB7 displayed similar growth in ethanol, BY-P41K-RPB7 was denoted as the control in the following experiments. Since yeast cell with *RPB7* null mutation is inviable, the control here bore native *RPB7* gene to neutralize the influence and interference caused by plasmid and chromosomal copies of *RPB7*.

### Cross-tolerance of M1

**Fig. 3:**
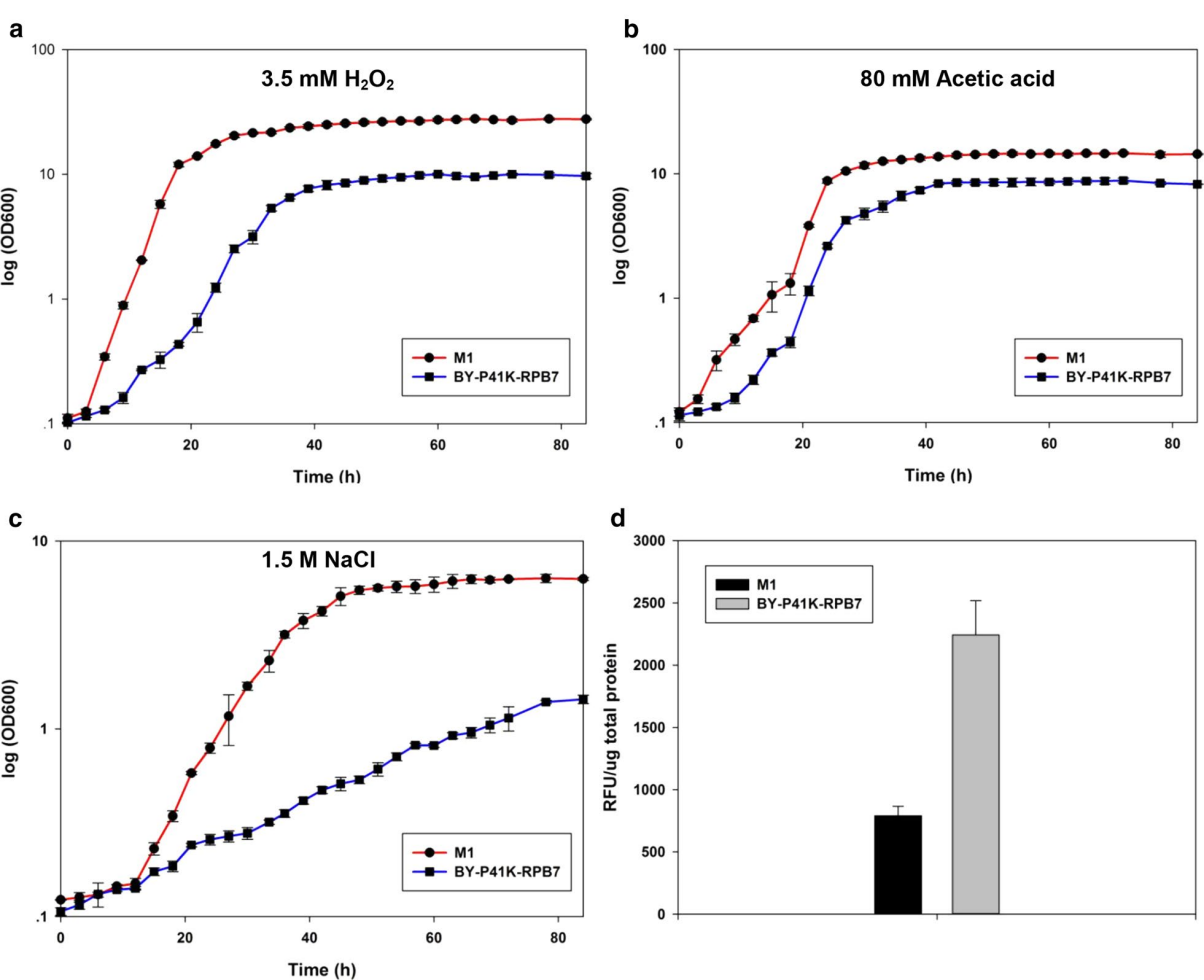
Cross-tolerance towards different inhibitors. M1 in **a** 3.5 mM H_2_O_2_; **b** 80 mM acetic acid; **c** 1.5 M NaCl. **d** Intracellular ROS level in M1 when cells reached early log phase (OD_600_ = 1). ROS level is positively correlated to the fluorescence intensity of probe H_2_DCFDA. All experiments were performed in triplicates

Previous studies have shown that intracellular ethanol may denature proteins and generate reactive oxygen species (ROS), which inhibit glycolysis, amino acid transport pathways and mitochondrial function, and impose oxidative damage to cellular lipids, proteins, and DNA [13, 17]. Since H_2_O_2_ is a typical stressor for testing strain oxidative stress tolerance [23, 44], M1 was subjected to H_2_O_2_ to test its oxidative stress resistance. As expected, M1 showed better growth than the control (Fig. 3a). In addition, we have also examined M1 tolerance towards acetate and NaCl, as acetate is the byproduct from ethanol fermentation and NaCl could lower the maximum specific growth rate of *S. cerevisiae* during fermentation [5, 50]. M1 exhibited enhanced resistance towards 80 mM acetic acid (Fig. 3b) and 1.5 M NaCl (Fig. 3c).

### Intracellular ROS level

As intracellular ROS level was an indicator of cell resistance towards toxic substances, it was also investigated in M1 [16]. M1 seems to have stronger capability in scavenging intracellular ROS as the intracellular ROS level of M1 was only ~37% of that of the control when cells were collected at early log phase (Fig. 3d).

### Inhibitors from lignocellulose hydrolysates

**Fig. 4:**
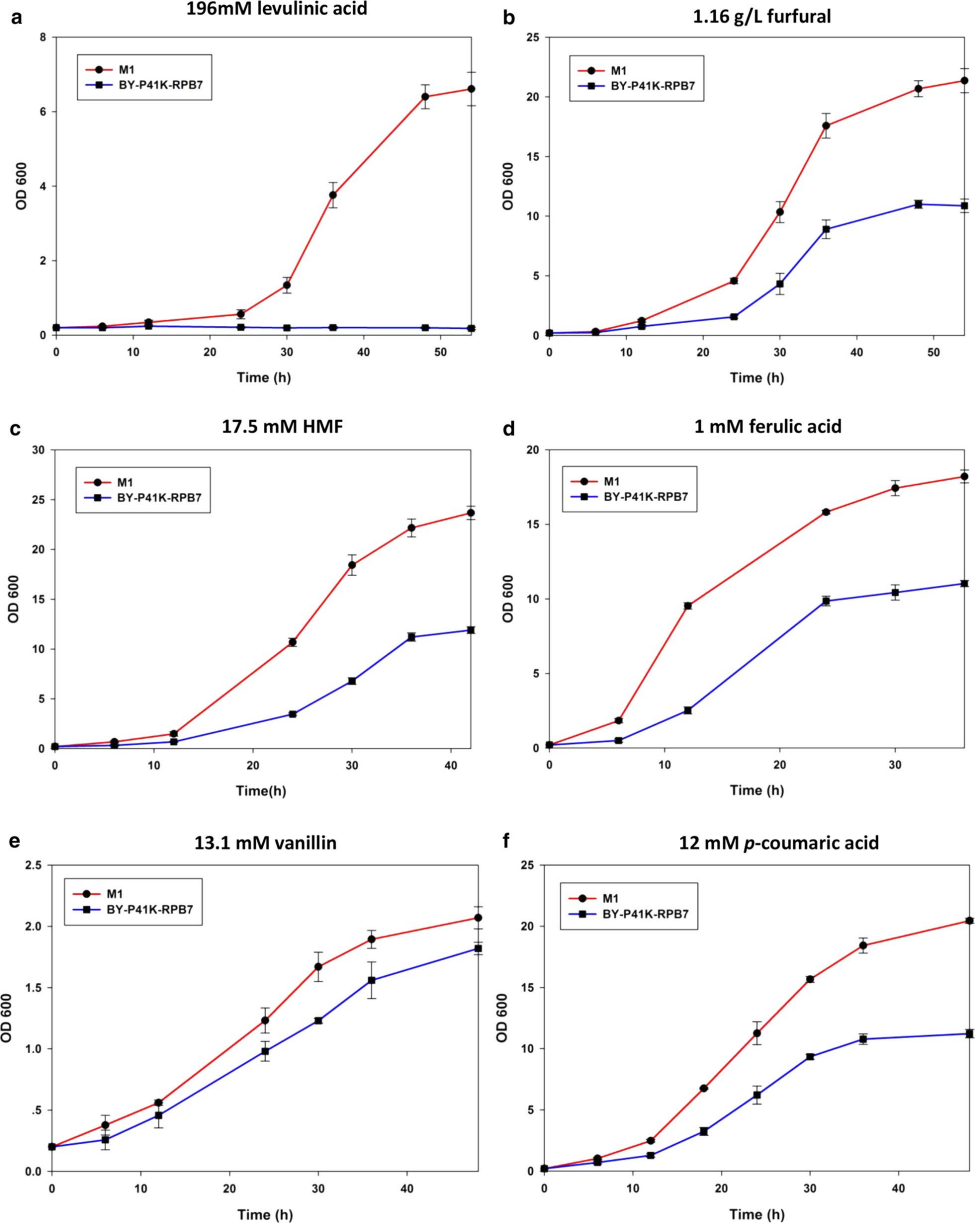
Cross-tolerance towards inhibitors from lignocellulose hydrolysates. M1 in **a** 196 mM levulinic acid; **b** 1.16 g/L furfural; **c** 17.5 mM HMF; **d** 1 mM ferulic acid; **e** 13.1 mM vanillin; **f** 12 mM *p*-coumaric acid

Moreover, M1 also exhibited cross-tolerance towards inhibitors from lignocellulose hydrolysates during bioethanol fermentation, including levulinic acid, furfural, HMF, and phenolic compounds (ferulic acid, vanillin, *p*-coumaric acid). When challenged by these inhibitors, M1 displayed better growth as compared with the control (Fig. 4).

### Ethanol fermentation

**Fig. 5:**
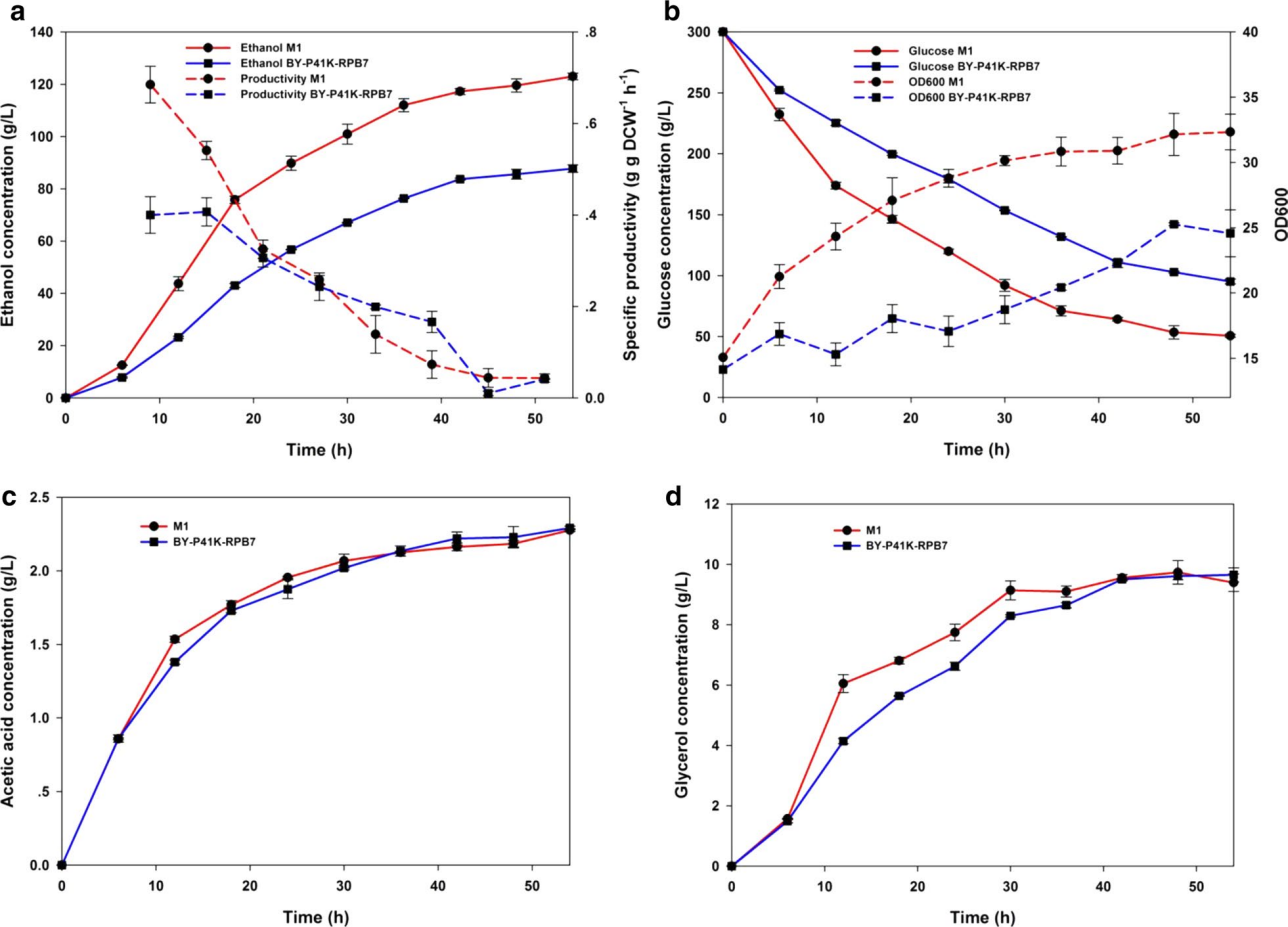
Fermentation characteristics during laboratory VHG fermentation. **a** ethanol concentration (*solid line*) and specific productivity (*dashed line*); **b** glucose concentration (*solid line*) and OD600 (*dashed line*); **c** acetic acid profile; **d** glycerol profile. Cells were cultured in biological replicates in 300 g/L glucose with a high inoculum of initial cell density of OD600 = 15 (~6 g DCW/L). Metabolites were analyzed by HPLC. *Specific productivity is expressed by ethanol productivity per viable cell following the equation below [29]: $\frac{{{\text{EtOH}}_{t} - {\text{EtOH}}_{t - 1} }}{{\left( {\frac{{{\text{DCW}}_{{{\text{viable}},t}} + {\text{DCW}}_{{{\text{viable}},t - 1}} }}{2}} \right)(t - t_{ - 1} )}}\left[ {{\text{g}} \cdot {\text{g DCW}}^{ - 1} \cdot {\text{h}}^{ - 1} } \right]$

VHG laboratory fermentation with high-cell-density and high-glucose cultures under micro-aerobic condition was applied to M1, mimicking industrially relevant conditions [6]. With an initial inoculum of OD600 ~15 (~6 g DCW/L) and 300 g/L glucose supply, the fermentation performance of M1 exceeded that of the control by showing better ethanol accumulation and productivity (Fig. 5a), rapid glucose consumption rate and improved biomass production (Fig. 5b). Ethanol titers in M1 reached 122.85 g/L after 54 h, approximately 96.58% of the theoretical yield (0.51 g/g glucose), ~40% increase as compared to that of the control (87.75 g/L). M1 also displayed ~33% increase in specific productivity (0.541 g g/DCW h) over the control (0.407 g g/DCW h) during the initial 12 h of fermentation. As shown in Table 1, M1 displayed higher cell density (+65.94%) and ethanol productivity (+39.99%) after 54 h, surpassing that of the control. On the other hand, M1 showed similar level of byproduct acetic acid and glycerol as the control during fermentation (Fig. 5c, d).

**Table 1:** Fermentation profiles of M1 and control after 54 h

Parameter	M1	Control	Percent improvement
Initial DCW (g/L)	6.02	5.66	–
Final DCW (g/L)	12.94 ± 0.54	9.83 ± 1.08	+65.94 ± 16.08%
Ethanol titers (g/L)	122.85 ± 1.46	87.75 ± 1.30	+39.99 ± 1.84%
Ethanol yield^a^	96.58 ± 1.14%	83.99 ± 1.24%	+14.99 ± 1.51%

^a^Ethanol yield is expressed as percentage of the maximum theoretical ethanol yield (0.51 g ethanol per gram of glucose consumed)

Fermentation was further investigated by varying initial glucose concentration, culturing pH, and host strain background. M1 demonstrated improved glucose-ethanol conversion rate in the range of initial glucose concentration tested (see Additional file 1: Figure S1). On the other hand, the optimum initial pH was found to be 7 for VHG fermentation (Additional file 1: Figure S2). Therefore, we chose pH 7 and initial glucose concentration at 300 g/L as fermentation condition.

**Fig. 6:**
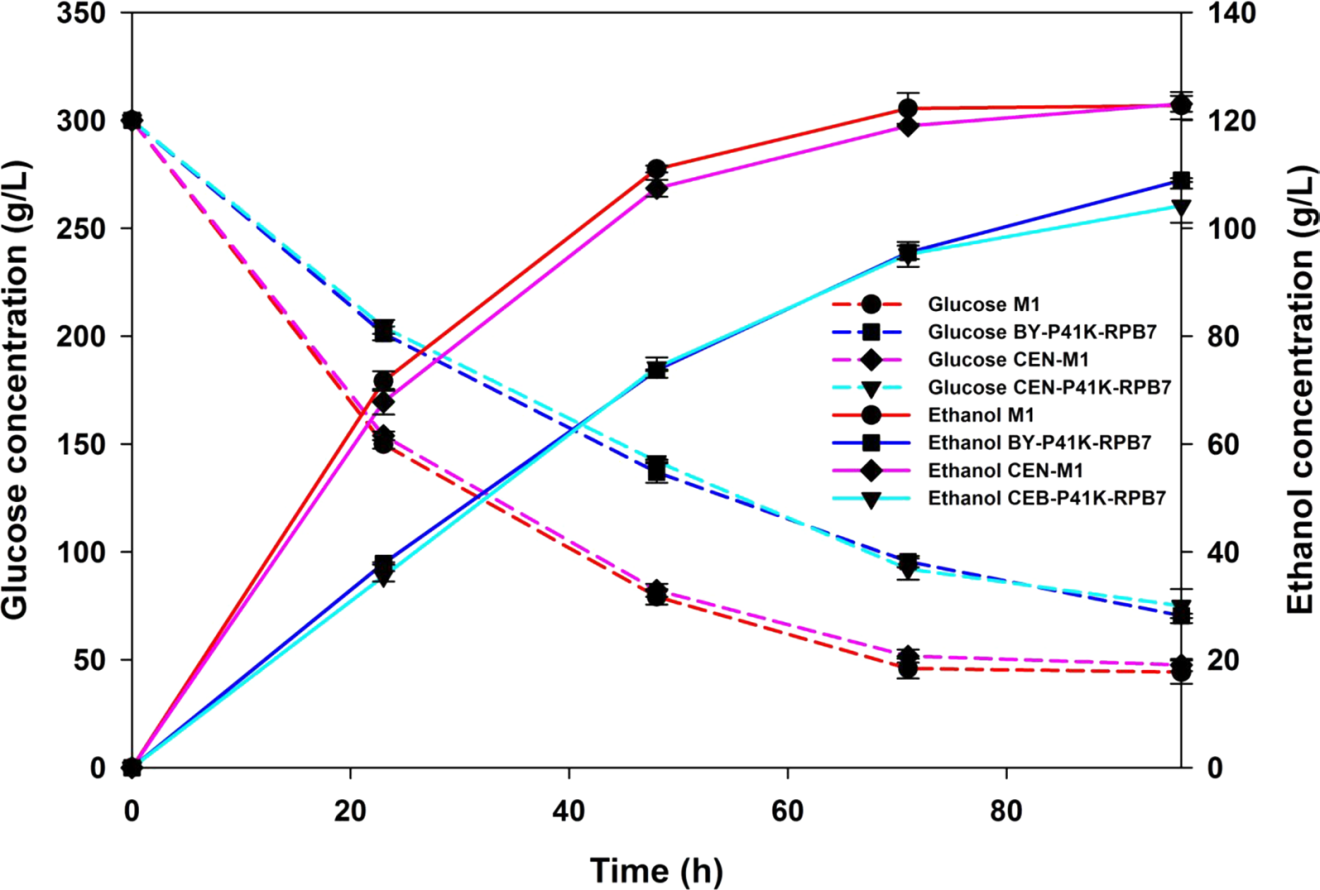
Fermentation characteristics. Ethanol production (*solid*) and glucose consumption (*dashed*) of M1 in CEN.PK and BY strains. CEN.PK2-1C strains containing mutated and native operon of p41K-*RPB7* were denoted as CEN-M1 and CEN-P41K-RPB7, respectively

In order to study M1 ethanol fermentation characteristics in other yeast strain background, plasmid p41K-*RPB7* from M1 and its native version were also transformed into strain CEN.PK2-1C, whose family strains are more prone to industrial strains [35]. The recombinants with mutated and native version of p41K-*RPB7* were denoted as CEN-M1 and CEN-P41K-RPB7, respectively. As shown in Fig. 6, CEN-M1 and M1 exhibited similar fermentation properties. CEN-M1 also displayed higher ethanol accumulation (~18.2%) and faster glucose consumption (~12.5%) as compared to its control (CEN-P41K-RPB7) after 96 h of VHG fermentation.

### DNA microarray assay and qRT-PCR results

Genome-wide DNA microarray assay was carried out to quantify global transcription changes in M1. The mRNAs from M1 and the control were extracted after 12 h VHG fermentation when M1 ethanol productivity was at its peak. Among the 6256 genes tested, 369 genes were observed with differential expression (>twofold, *p* value <0.05) in M1, including 144 genes up-regulated and 225 genes down-regulated. All raw data are available from Gene Expression Omnibus (GEO, http://www.ncbi.nlm.nih.gov/geo/), with the accession number of GSE77853.

**Fig. 7:**
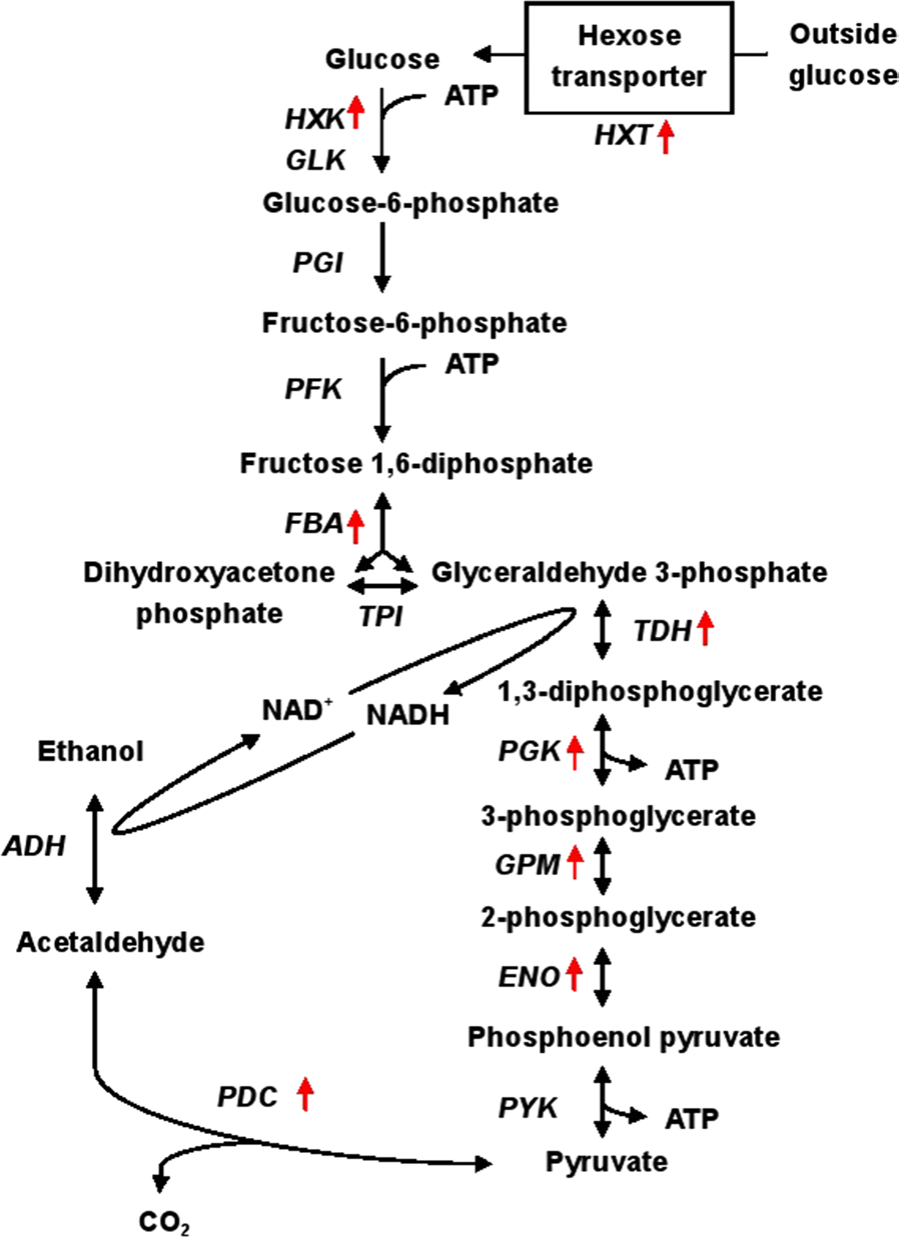
Gene expression level changes in ethanol synthesis pathway. Up-regulated genes (*red arrows*) from M1 in ethanol synthesis pathway

DNA microarray revealed that, among the 144 up-regulated genes, a significant set of gene are associated with energy metabolism, including glycolysis, alcoholic fermentation, hexose transport, and NAD^+^ synthesis. Specifically, among the ten enzymes involved in glycolysis, the following was found up-regulated, including hexokinase isoenzyme (*HXK1*, +twofold), fructose 1,6-bisphosphate aldolase (*FBA*, +2.04-fold), glyceraldehyde-3-phosphate dehydrogenase (*TDH1*/*2*/*3*, +2.10- to 2.21-fold), 3-phosphoglycerate kinase (*PGK1*, +2.49-fold), tetrameric phosphoglycerate mutase (*GPM1*, +2.02-fold), and enolase (*ENO1*/*2*, +2- to 2.47-fold) (Fig. 7). In the alcoholic fermentation pathway, increased induction of two pyruvate decarboxylase encoding genes (*PDC1* and *PDC5*) was also observed. In addition, genes responsible for hexose transportation, *HXT2*/*4*/*6*/*7* and de nova synthesis of NAD^+^, *BNA1*/*4*/*5* were up-regulated by 2- to 4-fold in M1.

Apart from genes involved in energy metabolism, the transcriptional reprogramming in M1 was quite broad, but it still demonstrated some enrichment of certain functional classes (Table 2). Oxidative stress response genes, such as *TSA1* (+2.16-fold), *TSA2* (+4.91-fold), *SOD1* (+2.15-fold) displayed elevated expression. Genes involved in the long-chain fatty acids metabolism, including *ACC1*, *FAS1*, *FEN1*, *OLE1*, and *SUR4*, also showed up-regulation in M1 (+2.2- to 3-fold). Another group with enhanced expression is associated with sterol synthesis, namely *ERG4*, *ERG20*, *HES1*, *UPC2* (+2- to 2.8-fold). While the major down-regulated genes (>twofold, *p* value <0.05) are from various functional groups, *e.g.*, biosynthesis of pyrimidines (*URA1*, *URA2*, *URA4*), helicase activity (*RRP3*, *YRF1*-*2*/*6*/*7*, *YIL177C*), and DNA mismatch repair (*MUS81*, *HOP2*, *MSH1*). Those genes that are sensitive to nitrogen catabolite repression were also down-regulated in M1 (*DAL3*, *DUR1,2*, *DUR3*, *DAL2*).

**Table 2:** Differentially expressed genes in M1 after 12-h VHG fermentation

Function group	Genes	Log 2 fold change*
*Up*-*regulated genes*		
Glucose, energy metabolism, and ethanol pathway	*BNA1*	4.08
	*PDC5*	3.63
	*PGK1, PDC1, HXT14, HXT6, HXT2, HXT7, BNA4, ENO1, BNA5, TDH1, TDH2, TDH3, ENO2, ALD4, FBA1, GPM1, HXK1*	2–3.1
Oxidative stress	*TSA2*	4.91
	*YHB1, TSA1, SOD1*	2–2.5
Fatty acids synthesis	*ACC1, FAS1, FEN1, OLE1, SUR4*	2.2–3
Cell wall synthesis and stability	*PIR1, YPS1, BAG7, GSC2, PSA1, CCW12, YLR194C*	2.5–3.5
Mental ion homeostasis	*CTR1, SRO77, VMA3, IZH4, TIS11*	2–3.1
ER-associated trafficking	*DFM1, GET3, CWH41*	2–2.8
Sterol synthesis	*HES1, ERG4, ERG20, UPC2*	2–2.8
*Down*-*regulated genes*		
Biosynthesis of pyrimidines	*URA1, URA2*	5.4–5.6
	*URA4*	2.56
Transcription regulators	*GCN4, RRN7, SMP1, YAP5, SAS2, RBA50, RRN6, IME1*	2–2.7
Helicase activity	*RRP3, YRF1*-*3, YRF1*-*6, YRF1*-*2, YRF1*-*7, YRF1*-*8, YIL177C, YHR219* *W, YML133C*	2–2.5
DNA repair	*MSH1, HOP2, MUS81*	2–2.3
Ribosome assembly and function	*RPS9B, RPF2, NSA3, YTM1, NOP6, NOP14, DRS1*	2–3
Sensitive to nitrogen catabolite repression	*DAL3, DUR1,2*	3.8–4
	*DUR3, DAL2*	2–3

* All fold changes were significant, with *p* value <0.05

Twelve genes were selected for qRT-PCR to validate the consistency of microarray results (Additional file 1: Table S3).

## Discussion

Inspired by the advantage of gTME in bringing along high degree of pleiotropy efficiently [27], we directly engineered *S. cerevisiae* RNAP II subunit Rpb7 and successfully isolated an ethanol-tolerant mutant via error-prone PCR. In accord with the assumption based on the correlation between cell viability and fermentation capability, M1 was also observed with improved ethanol titer of 122 g/L, which was comparable to the highest reported ethanol titer (117.6 g/L) from its parental strain S288c in complex media with 33% (w/v) glucose supply [37]. Moreover, M1 also demonstrated improved osmotolerance and resistance towards byproduct acetic acid, which are valuable traits for industrial ethanol fermentation [5]. M1 also showed improved growth in the presence of lignocellulose hydrolysate inhibitors, indicating its potential in second generation bioethanol fermentation.

The two amino acid mutations of M1, Y25N and A76T, locate at the N-terminal of Rpb7, which could interact with the core of RNAP II and bind single-stranded RNA in transcription [14]. A76T is located at a conserved β-strand of the ribonucleoprotein (RNP)-like domain of Rpb7, forming a highly conserved ‘tip loop’ to interact with CTD linker of subunit Rpb1 & Rpb6 and nascent RNA [4, 10, 41]. Mutation Y25N lies within the α-helix K2 of RNP, which also has putative RNA binding activity [33].

According to DNA microarray results, four high-affinity hexose transporter encoding genes (*HXT2*, *HXT6*, *HXT7*, *HXT14*) were up-regulated in M1, which assist glucose transport across plasma membrane, the first and rate-limiting step of glucose metabolism [36]. In addition, some genes involved in glycolysis pathway were also elevated in M1. The up-regulation of these genes may lead to faster glucose consumption in M1. Regarding the alcoholic fermentation pathway, qRT-PCR results showed that the expression of *PDC1* and *PDC5*, which encode pyruvate decarboxylase, the key enzyme in ethanol synthesis, were both elevated by twofold. Enzyme assay had also demonstrated that the activity of PDC in M1 was 28.3% higher than that of the control (Additional file 1: Figure S3), indicating glycolytic flux into ethanol formation [39]. The observed overexpression of genes (*BNA1*, *BNA4*, *BNA5*) involved in de nova synthesis of NAD^+^ from tryptophan may support the supplement of NAD^+^/NADH pool for boosted glycolysis and alcoholic fermentation [49]. Hence engineering Rpb7 might stimulate spontaneous metabolism adjustment in the complex metabolic network.

The cross-tolerance conferred by M1 towards all these inhibitors suggested synergistic mechanism in response to fermentation challenges. DNA microarray results showed up-regulated expression in genes associated with cell oxidative stress response, including thioredoxin peroxidase *TSA1* (+2.15-fold) and *TSA2* (+4.9-fold), nitric oxide oxidoreductase *YHB1* (+2.44-fold) and cytosolic copper-zinc superoxide dismutase *SOD1* (+2.15-fold). In particular, thioredoxin peroxidase is a well-known cellular antioxidant [51] that fights against cellular ROS toxicity in yeast. In addition, the transcription profile revealed that a set of up-regulated genes in M1 were related to sterol and fatty acids metabolism, which were involved in ethanol stress defense. For example, it was demonstrated before that increased ergosterol content could improve ethanol tolerance by strengthening yeast membrane structure [48]. M1 also demonstrated elevated genes expression in ergosterol synthesis pathway, i.e., *ERG20* (farnesyl pyrophosphate synthetase), *ERG4* (C-24(28) sterol reductase), and sterol synthesis regulation (*HES1*, *UPC2*). Previous work has shown that increased C18:1n-9 level could improve yeast ethanol tolerance [26] by reducing the fluidizing effects from ethanol [20]. Consistent with this finding, genes involved in the de novo biosynthesis of oleic (C18) acyl-CoA (C18:1n-9 precursor), such as *ACC1* (acetyl CoA Carboxylase I), *FAS1* (fatty acid synthetase subunit), and *FEN1* (fatty acid elongase) were also up-regulated in M1.

## Conclusions

This work is the first to demonstrate that eukaryotic RNAP II enzyme could be an alternative gTME target in eliciting improved phenotype, which is probably also applicable to other eukaryotes. The increased ethanol titers in M1 and elevated expression of genes involved in ethanol production pathway indicate that it is possible to engineer RNAP II to change the expression of multiple genes simultaneously to enhance the yield of desired products.

## Additional file

**Additional file 1: Table S1.** Primers used in plasmid construction and error-prone PCR. **Table S2.** Primers used in qRT-PCR. **Table S3.** Comparison between DNA microarray and qRT-PCR results on selected genes from M1 after 12h VHG fermentation. **Figure S1.** Ethanol profile with varying initial glucose supply under VHG fermentation. **Figure S2.** Ethanol profile with varying initial pH under VHG fermentation. **Figure S3.** PDC activity in M1 and the control after 12h VHG fermentation.

## Abbreviations

RNAP II: RNA polymerase II
VHG: very high gravity
gTME: global transcription machinery engineering
TF: transcription factor
CRP: cAMP receptor protein
qRT-PCR: quantitative real-time reverse transcription PCR
ROS: reactive oxygen species
