# Supplementary material for: Improving Saccharomyces cerevisiae ethanol production and tolerance via RNA polymerase II subunit Rpb7
Source: Biotechnol Biofuels. 2017 May 15;10:125. doi: 10.1186/s13068-017-0806-0 (PMC5433082; doi:10.1186/s13068-017-0806-0)
Supplement: Supplementary file 1 — Additional file 1: Table S1. Primers used in plasmid construction and error-prone PCR. Table S2. Primers used in qRT-PCR. Table S3. Comparison between DNA microarray and qRT-PCR results on selected genes from M1 after 12h VHG fermentation. Figure S1. Ethanol profile with varying initial glucose supply under VHG fermentation. Figure S2. Ethanol profile with varying initial pH under VHG fermentation. Figure S3. PDC activity in M1 and the control after 12h VHG fermentation. [file 13068_2017_806_MOESM1_ESM.docx]

**Improving** ***Saccharomyces cerevisiae* ethanol production and tolerance *via* RNA Polymerase II subunit Rpb7**

Zilong Qiu, Rongrong Jiang*

**PDC activity assay**

Cell extract was prepared by ultrasonication with 0.6 mm diameter glass beads at 0°C for 2 min with an ultrasonic processor (UP200S, Hielscher, Germany) following Flikweert’s approach (Flikweert et al., 1996). Pyruvate decarboxylase activity was assayed at 25 °C by monitoring the rate of the pyruvic-acid-dependent oxidation of NADH (340 nm), with ADH as a coupling enzyme using a Shimadzu UV-1800 spectrometer (Shimadzu Inc., Kyoto, Japan). The assay mixture (1 mL) consisted of: 40 mM pH 6.5 imidazole-HCI buffer, 0.15 mM NADH, 0.2 mM-thiamine pyrophosphate, alcohol dehydrogenase 88 U mL^-1^ (Sigma Aldrich, St. Louis, MO, USA), 5 mM MgCl, and cell free extract. The reaction was initiated by the addition of 50 mM pyruvate. Enzyme activity was normalized by total protein concentration measured by Braford assay.

[Flikweert MT](http://www.ncbi.nlm.nih.gov/pubmed/?term=Flikweert%20MT%5BAuthor%5D&cauthor=true&cauthor_uid=8904337), [Van Der Zanden L](http://www.ncbi.nlm.nih.gov/pubmed/?term=Van%20Der%20Zanden%20L%5BAuthor%5D&cauthor=true&cauthor_uid=8904337), [Janssen WM](http://www.ncbi.nlm.nih.gov/pubmed/?term=Janssen%20WM%5BAuthor%5D&cauthor=true&cauthor_uid=8904337), [Steensma HY](http://www.ncbi.nlm.nih.gov/pubmed/?term=Steensma%20HY%5BAuthor%5D&cauthor=true&cauthor_uid=8904337), [Van Dijken JP](http://www.ncbi.nlm.nih.gov/pubmed/?term=Van%20Dijken%20JP%5BAuthor%5D&cauthor=true&cauthor_uid=8904337), [Pronk JT](http://www.ncbi.nlm.nih.gov/pubmed/?term=Pronk%20JT%5BAuthor%5D&cauthor=true&cauthor_uid=8904337). Pyruvate decarboxylase: an indispensable enzyme for growth of Saccharomyces cerevisiae on glucose. Yeast. 1996; 12**:** 247-57.

**Table S1** Primers used in plasmid construction and error-prone PCR

| Primer | Sequence |
| --- | --- |
| 1 | GCAGGCGGATCCCTCAATTTCTGAGAATGTT |
| 2 | GCGCCGGAATTCCAAGTGAT TAAATAGCACC |
| 3 | C GAGCTC CGGGGTTCTTCGGGCCAAATGAA |
| 4 | C GGATCC TCTCAGAAATTGAGTTATTTATA |
| 5 | CCGTTTCTCCTCCTACACCATTCT |
| 6 | GGACCTAGACTTCAGGTTGTCTAA |

**Table S2** Primers used in qRT-PCR

| Primer | Sequence |
| --- | --- |
| *URA3*-F | GTGGTGGGCCCAGGTATTGTTA |
| *URA3*-R | CACCGGGTGTCATAATCAACCA |
| *URA1*-F | CCATGGGTTTACCAAACGAAGG |
| *URA1*-R | ACTTGTGGTTTCCCAGGCACAT |
| *URA2*-F | TGGGAGGTTTAGGTTCCGGTTT |
| *URA2*-R | AGATGGGGCTACAACGATGGAA |
| *RND18*-F | TTAATGACCCACTCGGCACCTT |
| *RND18*-R | GGTGAGTTTCCCCGTGTTGAGT |
| *FBA1*-F | GCCGCTATGGACCAATGGTTAG |
| *FBA1*-R | GCGATGTCACCAGCGTACAAAC |
| *MDH2*-F | CGGTGGTGATGAAGTGGTCAAG |
| *MDH2*-R | TGGGGAAGTTGTTCGCATCTTT |
| *TDH3*-F | CAGCTAACTTGCCATGGGGTTC |
| *TDH3*-R | ATGGAGCCAAACAGTTGGTGGT |
| *ITR1*-F | TAAACCCGTGAACGACGAGGAT |
| *ITR1*-R | GGTAGCCGCAGTGACAATTTCC |
| *ENO2*-F | CCTTCGCTGAAGCCATGAGAAT |
| *ENO2*-R | TCGTGACCAGCAGCCTTGATAG |
| *PDC1*-F | AAGGAAGCCGTTGAATCTGCTG |
| *PDC1*-R | TGGGACAGCAACTGGCTTGTAA |
| *PDC5*-F | CGCTCCAGCTGAAATTGACAGA |
| *PDC5*-R | GAAGCACAAGCATCAGCCAAGA |
| *ERG20*-F | GGTTGGTGCATTGAGTTGTTGC |
| *ERG20*-R | GGTTTGGAAGGTGACCTCATGG |
| *FEN1*-F | TGTTCGAGCGTTATCCCCAAC |
| *FEN1*-R | AGGGGGCAAAGTGCTTAATGG |

**Table S3** Comparison between DNA microarray and qRT-PCR results on selected genes from M1 after 12h VHG fermentation.

| **Gene Symbol** | **DNA Microarray** | | **qRT-PCR** | |
| --- | --- | --- | --- | --- |
|  | **Log2 fold change** | ***p*-value** | **Log2 fold change** | ***p*-value** |
| *URA3* | +13.15578449 | 6.93E-04 | +10.479 | 0.0049 |
| *PDC5* | +3.629911 | 0.016687 | +1.826 | 0.000153988 |
| *ENO2* | +2.05134 | 0.001484 | +0.898 | 0.04774966 |
| *ERG20* | +1.010312659 | 5.78E-04 | +3.976 | 0.03387 |
| *FEN1* | +1.163127384 | 0.006871 | +0.801 | 0.045055 |
| *ITR1* | +1.048042232 | 4.46E-05 | +0.685 | 0.011993 |
| *PDC1* | +2.143487 | 0.047585 | +2.483 | 6.54026E-05 |
| *TDH3* | +2.108383 | 0.009277 | +2.033 | 0.015969 |
| *MDH2* | -1.240953431 | 0.004 | -1.682 | 0.06595 |
| *URA1* | -2.439799814 | 0.001739 | -2.133 | 0.007919 |
| *URA2* | -2.467311386 | 0.001129741 | -3.02 | 0.00598 |
| *FBA1* | +2.045623 | 0.020591116 | +1.011 | 0.005983 |


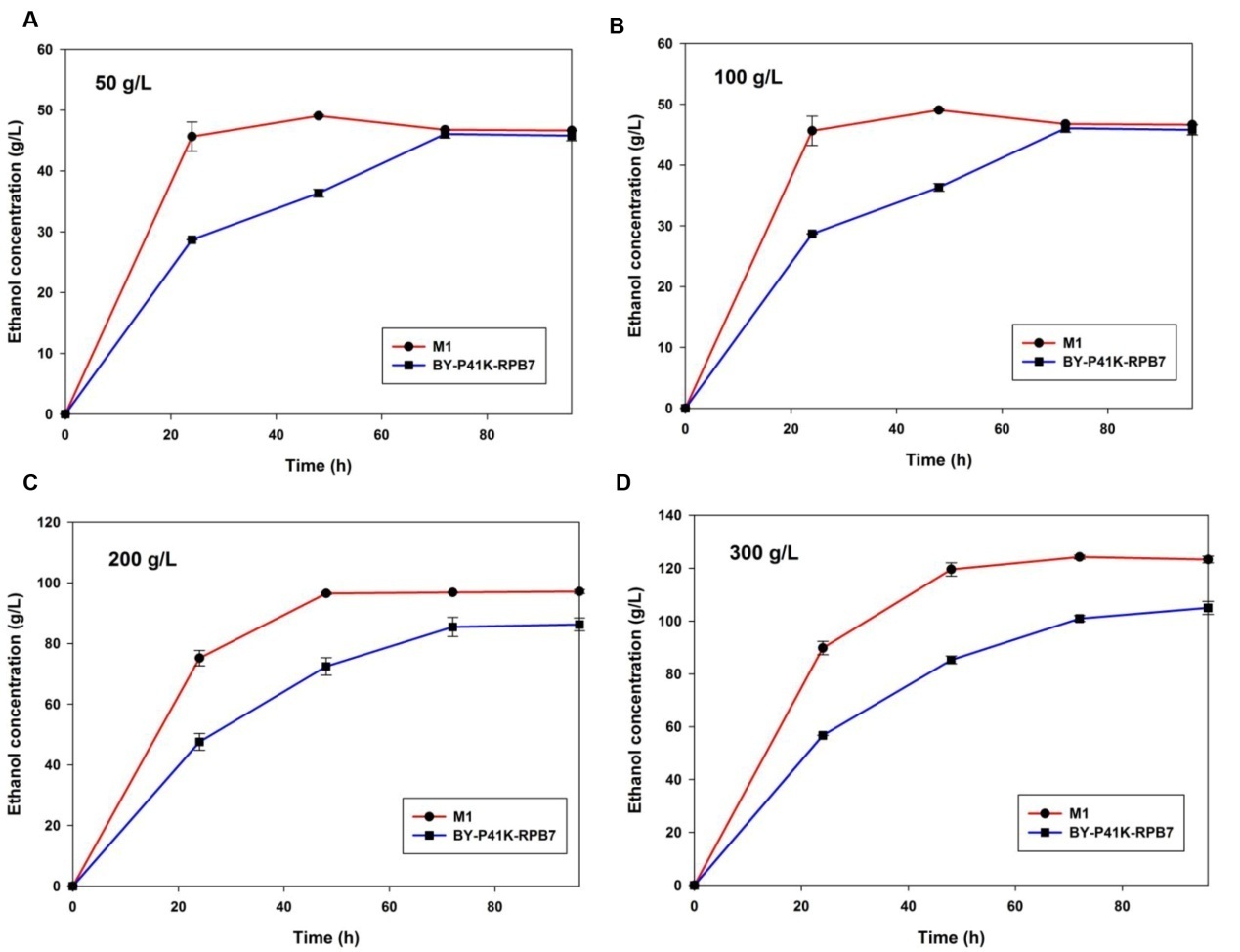


**Figure S1** Ethanol profile with varying initial glucose supply under VHG fermentation (A) 50 g/L glucose; (B) 100 g/L glucose; (C) 200 g/L glucose; (D) 300 g/L glucose.


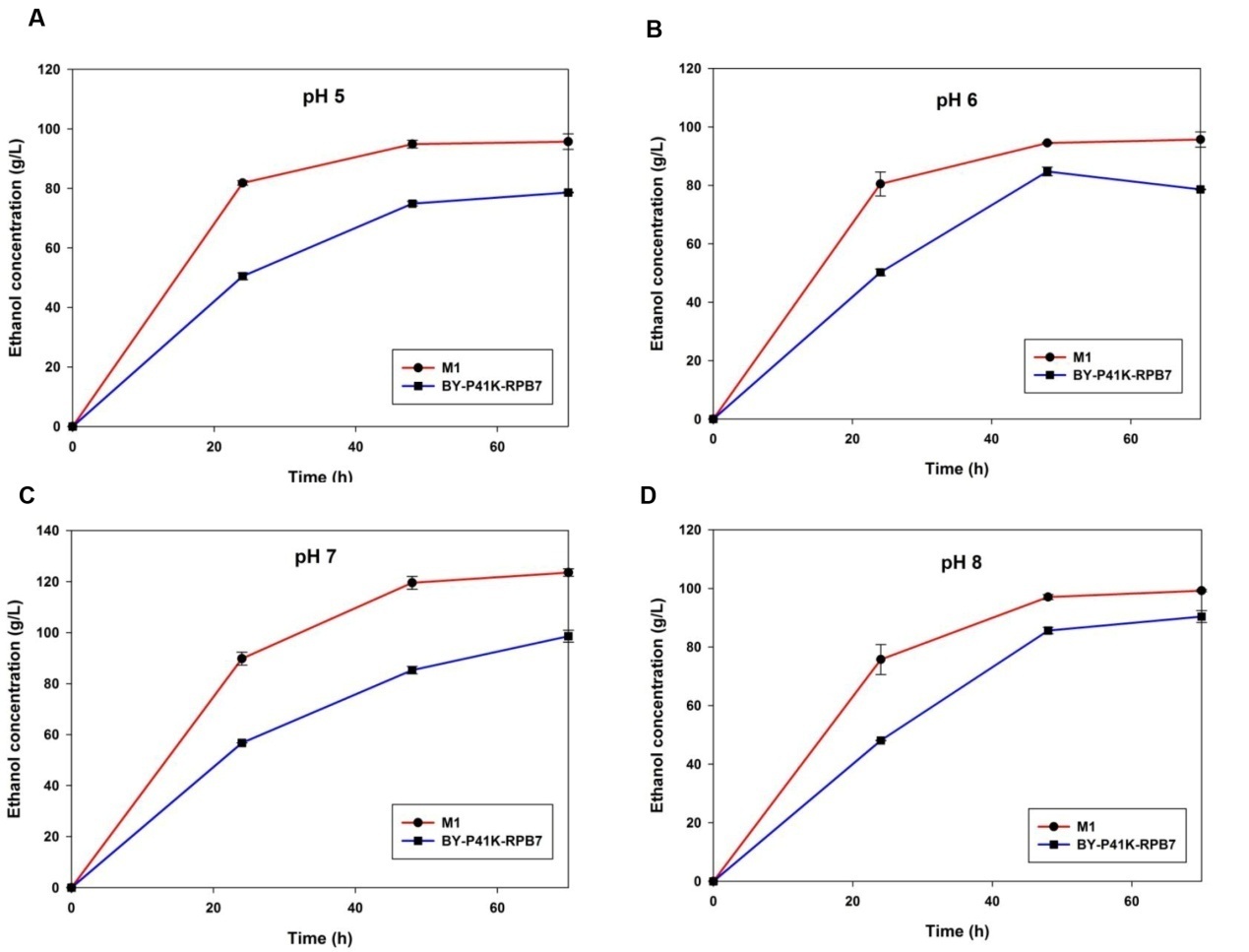


**Figure S2** Ethanol profile with varying initial pH under VHG fermentation. (A) pH 5; (B) pH 6; (C) pH 7; (D) pH 8.

**
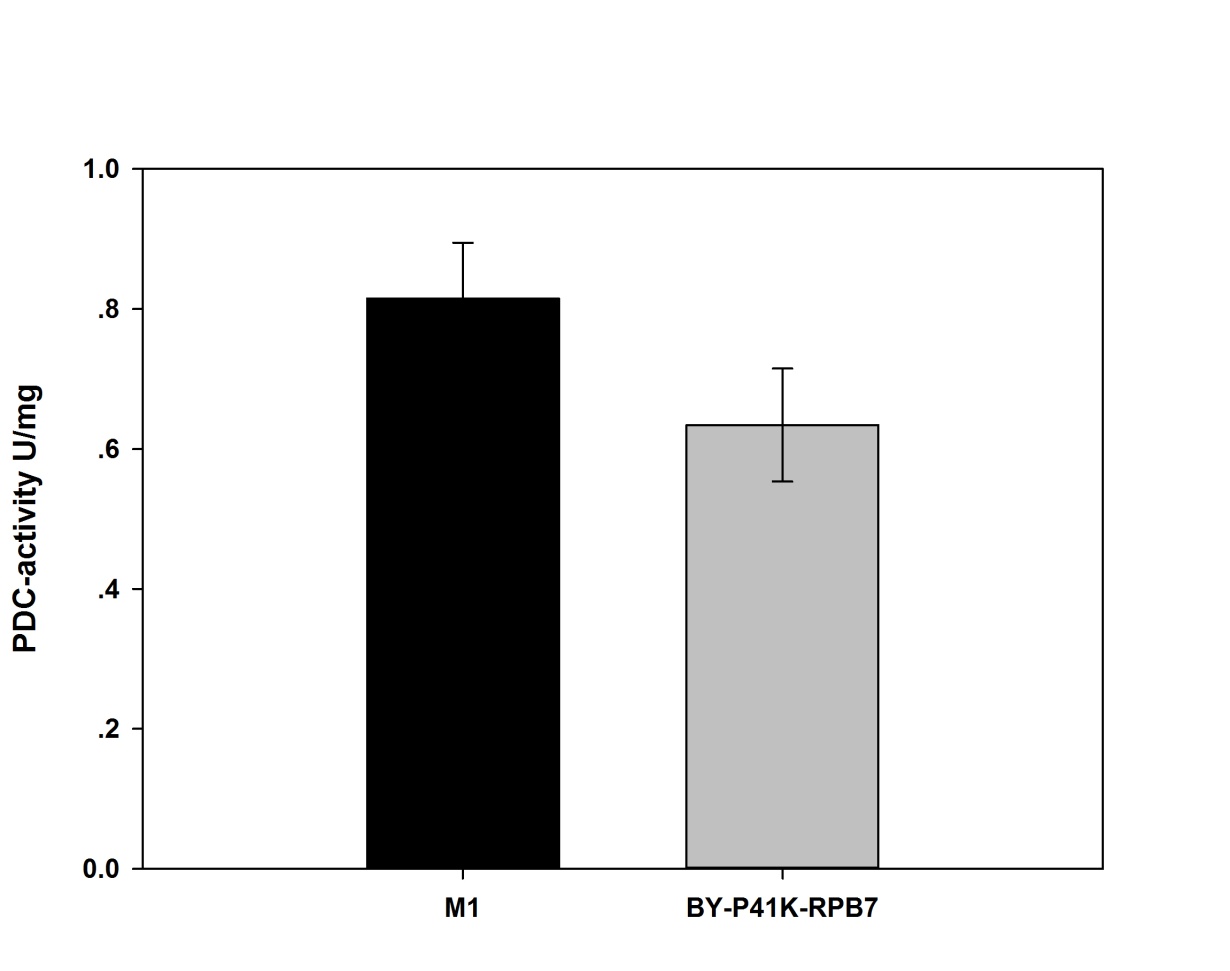
Figure S3** PDC activity in M1 and the control after 12h VHG fermentation.
